# Myiasis as a Rare Complication of Male Circumcision: A Case Report and Review of Literature

**DOI:** 10.1155/2012/483431

**Published:** 2012-09-20

**Authors:** Muhammad Rajib Hossain, Kazi Mazharul Islam, Junaid Nabi

**Affiliations:** ^1^Upazilla Health Complex, Bamna, Barguna 8700, Bangladesh; ^2^Surgery Unit-III, Department of Surgery, Dhaka Medical College Hospital, Dhaka 1000, Bangladesh; ^3^Surgery Unit-IV, Department of Surgery, Shaheed Suhrawardy Hospital, Dhaka 1207, Bangladesh

## Abstract

*Introduction*. Circumcision is a common procedure carried out around the world. Due to religious reasons, it is routinely done in Bangladesh, by both traditional as well as medically trained circumcisers. Complications include excessive bleeding, loss of foreskin, infection, and injury to the glans penis. Myiasis complicating male circumcision appears to be very rare. *Case Presentation*. In 2010, a 10-year-old boy presented to the OPD of Dhaka Medical College Hospital with severe pain in his penile region following circumcision 7-days after. The procedure was carried out by a traditional circumciser using unsterilized instruments and dressing material. After examination, unhealthy granulation tissue was seen and maggots started coming out from the site of infestation, indicating presence of more maggots underneath the skin. An emergency operation was carried out to remove the maggots and reconstruction was carried out at the plastic surgery department. *Conclusion*. There is scarcity of literature regarding complications following circumcision in developing countries. Most dangerous complications are a result of procedure carried out by traditional circumcisers who are inadequately trained. Incidence of such complications can be prevented by establishing a link between the formal and informal sections of healthcare to improve the safety of the procedure.

## 1. Introduction

Circumcision in Bangladesh is commonly conducted in neonates, infants, and children for religious reasons, in other parts of the world, it is carried out due to cultural influence and many are done for medical reasons. On an average, there is a prevalence of one in every three males being circumcised around the world; the demography, however, shows much variation [1]. Circumcision being an operative procedure comes with its share of complications as well [2]. Pain, swelling, bleeding, and inadequate skin removal are usually seen as early or intraoperative complications which are not serious and require minor interventions. There have been reports of serious complications during the operation as well, such as excessive bleeding which has resulted in death and amputation of the glans penis [3]. Complications which come under the postoperative or late category include infection, meatal stenosis, pain, urinary retention, fistulas, sexual dysfunction, and loss of penile sensitivity [4].

Myiasis—the infestation of live vertebrate animals with dipterous larvae [5] as a complication of circumcision—is rather a very rare complication encountered in clinical practice, and according to the databases, we searched that it has not been reported so far in the literature. Myiasis in dead bodies is fairly common, but this infestation is very less often seen in living tissues. Worldwide, the most common flies that cause the human infestation are *Dermatobia hominis* (human botfly) and *Cordylobia anthropophaga* (tumbu fly). These flies drop eggs containing larval stage on skin, wounds, or natural orifices of an immobile person, though recently described cases from many parts of the world have involved nasotrachea, mouth, brain, penis, gums, urinary bladder, and colon [6, 7].

We report a very rare complication following circumcision and the need for prevention of such cases to reduce the economical and psychological burden on the patients.

## 2. Case Report

A 10-year-old boy from Narayangonj, Bangladesh, presented to the Dhaka Medical College OPD in February 2010 with a 7-day history of pain in the penile region following circumcision. After thorough history, it was learnt that the operation was performed by an unqualified traditional “itinerant circumciser.”

It was mentioned by the parents that the procedure was carried out without proper instruments and sterilization. A tight bandage was also applied over the wound, which caused severe pain. A few days later, when the bandage was removed, the parents of the boy mentioned that maggots were coming out from the wound site. 

The boy was treated with hydrogen peroxide before being referred to the tertiary care hospital in Dhaka.

**Figure 1:**
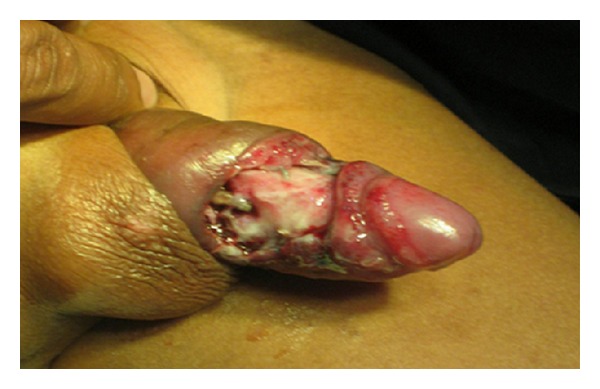
During examination of the penile shaft, several maggots were seen crawling out from underneath the skin.

On examination, the penis was grossly swollen, the penile skin was edematous, and erythema was also seen. There was a gap in the skin behind the corona glandis exposing the shaft. The base was covered with granulation tissue. After a little manipulation, maggots started to come out from underneath the skin of the shaft (Figure 1). The skin could not be pulled back. On palpation, it was observed that there were more maggots under the skin extending to the root of the penis.

**Figure 2:**
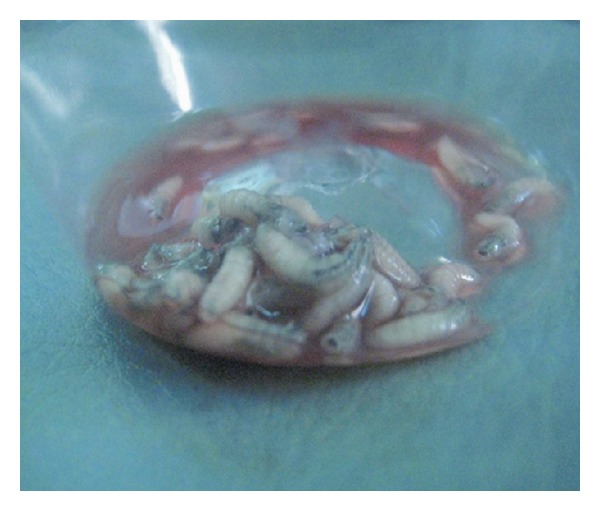
Postoperative picture showing maggots after removal from the infected site.

A decision for an immediate operative intervention was taken. Under a penile block, a dorsal slit was made on the penile skin and maggots started to come out in large numbers. There was some necrosed skin and multiple pockets containing maggots, some even extending to the mons pubis which made the exploration difficult. Some of these insects were removed by plane tissue forceps and some were treated with hydrogen peroxide to completely eradicate these pockets. There were almost 30 maggots which were removed and an antibiotic dressing was done (Figure 2). The pain subsided immediately following debridement. A day later, reexploration of the wound was done and remaining maggots came out spontaneously.

The patient was treated with antibiotic dressing and oral antibiotics for 30 days following the operation till there was assurance that the wound had healed properly. On final exploration of the site, the shaft including all three corpora and the glans were found to be healthy. Later, the patient was referred to the plastic surgery department for aesthetic management of the site. As the site was ready for graft, they made a partial thickness skin graft which was taken there without any complication.

## 3. Discussion

Even though male circumcision is a fairly regular procedure done in Bangladesh, there is very limited literature regarding the complications. 

The few epidemiological studies carried out in different parts of the world show that the most common complications are excessive blood loss and infection. 

We carried out a systemic review of studies regarding complications of circumcision [8] but did not encounter any incidences of myiasis following circumcision. We do realize, however, that for such cases to be documented a broader study is required, especially in the context of this country. We carried out a search in various databases under the terms “myiasis complicating male circumcision,” “complications of male circumcision,” and “myiasis complicating neonatal circumcision” but failed to retrieve any reports which led us to believe that myiasis appears to be an exceedingly rare complication of male circumcision.

It is interesting to note that frequency of complications and other adverse effects that occur among neonates and infants is relatively less as compared to older boys, even in similar settings, most probably because of the simpler nature of procedure in the young age group and the healing capabilities in the new born [9]. Also, a major advantage of neonatal circumcision is that suturing is not usually necessary and alternatives such as disposable clamps and cyanoacrylate glue can be used [10].

The complication reported appears to be a result of unsterilized instruments used by the circumciser and the use of an improper technique in handling of the wound. As Mattson et al. reported, the lack of basic instruments and inadequate supplies [11] as well as training in developing countries always raises concerns about the safety of carrying out these procedures. Steps such as taken by other developing countries like Ghana, where the formal Health Service provides training to traditional circumcisers in Accra about the basic hygiene and provision of necessary equipment including sterile gloves and dressing [12], can be followed in this country to ensure minimum complications.

## 4. Conclusion

The available literature on prevalence of complications following male circumcision in Bangladesh is scarce. There is also an immediate need to improve safety of the procedure through enhanced training for both traditional and medically trained circumcisers, especially in the context of this country where majority of the population still reside in medically underdeveloped rural areas. We can emulate the example of South African developing countries and make an attempt to reduce the occurrence of common as well as rare complications. Creating links between the formal and informal health sectors will likely result in improved safety of this common procedure.
